# Supplementary material for: Shared Treg dysregulation underlies psoriasis vulgaris and atopic dermatitis through the PHF19-PRC2 epigenetic axis
Source: Front Immunol. 2026 Aug 20;17:1932788. doi: 10.3389/fimmu.2026.1932788 (PMC13538097; doi:10.3389/fimmu.2026.1932788)
Supplement: Supplementary file 3 [file DataSheet3.zip › Additional files/Additional file 8/28.OPTN.leaveoneout.pdf]

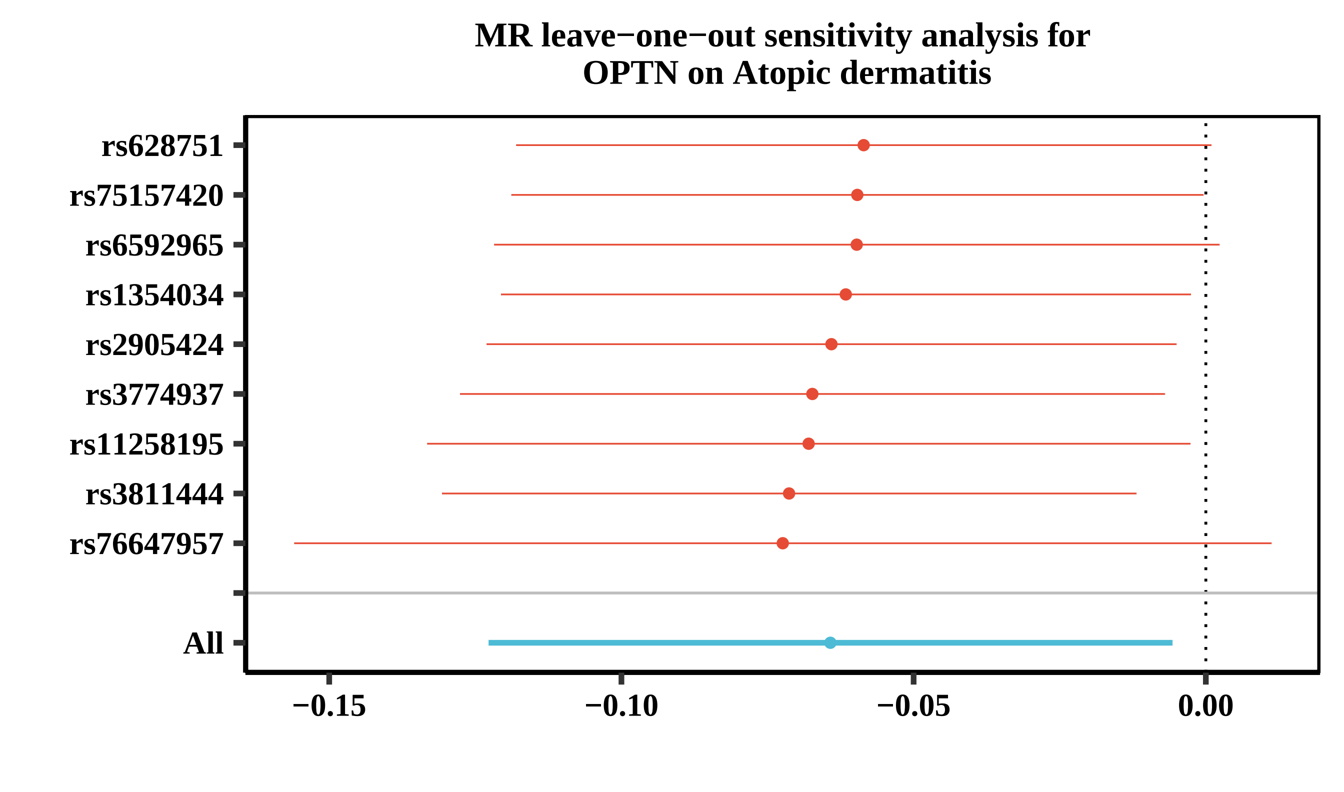
MR leave−one−out sensitivity analysis for
OPTN on Atopic dermatitis
rs628751
rs75157420
rs6592965
rs1354034
rs2905424
rs3774937
rs11258195
rs3811444
rs76647957
All
−0.15
−0.10
−0.05
0.00
